# “*Faith Is Not Enough?*” Ego-Resiliency and Religiosity as Coping Resources with Pandemic Stress—Mediation Study

**DOI:** 10.3390/ijerph20031942

**Published:** 2023-01-20

**Authors:** Roman Ryszard Szałachowski, Wioletta Tuszyńska-Bogucka

**Affiliations:** 1 Institute of Psychology, University of Szczecin, 71-415 Szczecin, Poland; 2 Department of Human Sciences, WSEI University, 20-209 Lublin, Poland

**Keywords:** religiosity, psychological resources, ego-resiliency, PTSD, depression, mediation model

## Abstract

Based on the concepts of Pargament’s adaptational functions of religiosity, Huber’s centrality of religiosity, and Block’s conceptualisation of ego-resiliency as psychosocial resources, a nonexperimental, moderated mediation project was designed for a group of 175 women and 57 men who voluntarily participated in an online study to determine whether and to what extent religiosity mediated or moderated the relationship between ego-resiliency and the severity of PTSD and depression during the COVID-19 epidemic. The analyses carried out showed that the studied variables, ego-resiliency and centrality of religiosity, were predictors of the intensity of some psychopathological reactions caused by the COVID-19 pandemic but were not connected via a mediation relationship. Therefore, one question remains open: what is the role of ego-resiliency and the nature of the stated immunogenic effect of the centrality of religiosity in dealing with the critical threat to mental health that is the COVID-19 pandemic?

## 1. Introduction

The situation of major global crises, such as the SARS-CoV-2 pandemic, makes it necessary to seek resources on which one can build resilience to prolonged situations of fear, uncertainty, and social isolation (because, perhaps, other pandemics are already waiting). In dealing with stress, the role of so-called psychosocial resources, which play an important role in the process of strengthening resilience and reducing the negative effects of stress, is currently emphasised [1].

One of the most important of these seems to be ego-resiliency. In the study, we chose the conceptualisation of Block and Block and their thirty years of work on ego-resiliency as a theoretical and methodological basis [2]. Block and Block define ego-resiliency as adaptive flexibility, the ability to adjust the level of impulse control (increase or decrease) to a given situation [2,3,4], and improving affective regulation processes [5], which helps people adapt to the situation and use other personal resources effectively [6,7]. It is seen as the ability to cope with significant difficulty and stress, and it is both a process and an outcome. Sometimes it is seen as a supporting function, ‘regressing’ to an earlier level of ability by overcoming barriers fostered by facilitating factors such as personality traits and environmental support. At other times, it is seen as the ability to integrate lifelong learning and expand coping repertoires as an active process incorporating positive adaptation within the context of serious harsh conditions [4,8,9,10,11,12], thus achieving new understanding that encompasses what has happened before but also extends beyond it. Resilience is the ability to access inner wisdom and strength reinforced by time and experience. It is one of the key psychosocial resources in situations including global tragedies, such as a pandemic [13]. In order to improve public mental health during this pandemic, accumulated knowledge on ego-resiliency can be used to provide practical solutions to help people cope effectively with the challenges [14]. An increasing body of empirical data suggests that religiosity serves as a factor of self-resilience. It constitutes individual identity and enables social networking, which in turn acts to facilitate effectiveness of coping with stress and other psychological conditions, i.e., depression and anxiety. Further research also indicates a correlation with well-being [15].

People with high levels of ego-resiliency are more likely to experience positive effects, are more self-confident, and have overall better psychological adaptivity while conserving their resources. It also increases resistance to stress [6,16,17,18,19,20]. The results of recent studies indicate that this is also a confirmed effect in the case of negative states associated with the COVID-19 pandemic [21,22], but its direct tonic effect on such experiences is not always confirmed [14]. However, the relationship between ego-resiliency and mental health during the COVID-19 pandemic has not been adequately explored [14]. Therefore, the purpose of this study was to examine the effect of ego-resiliency on mental health during the current pandemic. First, this study examined the effect of ego-resiliency on mental health.

Is the relationship between ego-resiliency and adjustment to the pandemic situation a simple one, or might other psychological variables play a role in the relationship between them? In order to define this group of factors, the term “resources conducive to coping with stress” is used. One possible resource with documented effects on health is religiosity, which is described as a type of meta-resource whose central position in an individual’s life results in religious content having an autonomous and wide-ranging impact on his or her overall experience and behaviour and thus also on mental health [23]. It seems to be an immunogenic factor usually reported in the context of more efficiently dealing with various types of burdens [24,25].

**Figure 1:**
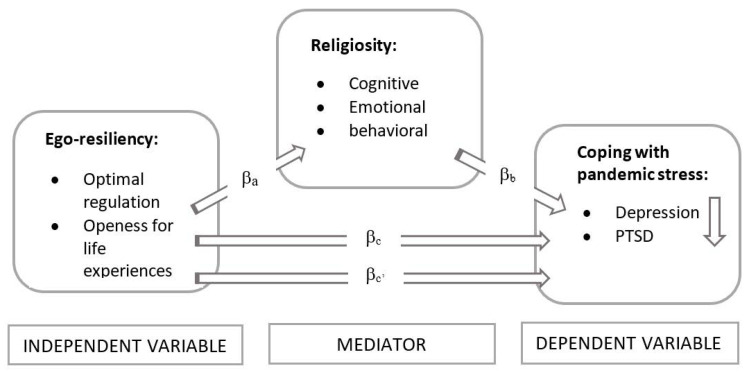
Theoretical model of the ego-resiliency–religiosity–coping connections.

What links religiosity to ego-resiliency, and both of these resources to health? There are both empirical and theoretical reasons to assume a positive relationship between these phenomena. A bridge between these areas may be Pargament’s theory, which emphasises the immunogenic role of religiosity in coping with stress [26,27]. Pargament emphasizes the role that religion may play in the complex process of resilience through which people attempt to understand and cope with the various problems that arise in their lives. Religious involvement may facilitate the development of meaning of the pandemic situation, which helps one to cope. Religiosity thus provides opportunities to find meaning in a difficult situation, and ego-resiliency activates resources and therefore coping strategies [27]. Religiosity would thus be a type of ‘access path to resources’ remaining in the ego-resiliency domain. The construct of a religious meaning system can also be recalled here. It emphasises the meaning-making and orienting function of religion [28,29,30] especially in difficult situations [31], placing religiosity in the stream of research on orienting and adaptive meta-resources of great significance for an individual’s health. We suppose that religiosity can explain the mechanism of the relationship between ego-resiliency and coping with pandemic stress (measured by the level of depression and PTSD connected with pandemic—Figure 1).

Which approach to understanding religiosity should be chosen in order to successfully complete the research project? It seems that from the perspective of the current knowledge and methodological requirements, the optimal model is one that assumes the multidimensionality of the construct of religiosity, ensures its operationalisation, and is equipped with reliable research instruments.

A systematic review of the concept shows that it has been understood in very different ways. In this paper, we treat religiosity in the classical psychological sense (In contrast to the theological or religious studies approach. Psychology in its paradigm does not study the factuality or nature of supernatural reality), i.e., as engaging in beliefs and practices characteristic of a given religious tradition [32]. Huber’s [33] dual approach to religiousness—i.e., psychological, as originally developed by Allport [34], and sociological, as developed by Glock and Stark [35]—led to the development of a phenomenologically complex model of religiousness that includes the following aspects: cognitive (religious beliefs and knowledge), emotional (religious feelings), and behavioural (religious practices). These aspects of religiosity seem to be beneficial when dealing with the stress of the global pandemic caused by COVID-19.

In conclusion, we can say, that religiosity (1) helps an individual formulate and adopt wider life perspectives; (2) gives each individual sense of an intrinsic energy, positivity, and direction; (3) serves as an escape mechanism and a buffer, the lack of which can result in mental disorders; (4) aids socially adjusted behaviours; (5) can serve as a safe outlet for those exhibiting mental disorders [36].

This article examines the role of ego-resiliency and the central role of religiosity in dealing with the psychopathological reactions caused by the pandemic. The authors expected both religiosity and ego-resiliency to be predictors of the severity of respondents’ psychopathological reactions (depression and PTSD) during the COVID-19 lockdown (H1). In addition, we expected that religiosity would enhance the positive (decreasing) effect of ego-resiliency on the level of psychological reactions (depression and PTSD) associated with the pandemic situation (H2).

## 2. Methods

### 2.1. Participants

The studies were carried out during a national lockdown (announced on 13 March). The studied sample (*N* = 232, *M*_AGE_ = 37.95, *SD* = 13.28) included 175 women (75.4%, *M*_AGE_ = 36.84, *SD* = 13.27) and 57 (24.5%, *M*_AGE_ = 41.39, *SD* = 12.82) men aged between 18 and 71 years. The survey was addressed to people all over Poland. Such contextualization is needed, as what “religiosity” is and how it influences individuals differs between different sociocultural contexts. The authors were looking for new relationships between studied variables. Table 1 presents the basic sociodemographic characteristics.

### 2.2. Measures and Procedures

The study used four standardised tools with satisfactory psychometric properties to measure ego-resiliency, centrality of religiosity, severity of PTSD, and depression:

The Polish version of the Ego-Resiliency Scale (ER89) of Block and Kremen in the adaptation of Kołodziej-Zaleska and Przybyła-Basista was used to measure the ego-resiliency trait understood as the ability of dynamic and proper self-regulation to enable faster adaptation to changing conditions. The original version of the questionnaire had a one-factor structure and contained 14 statements with a 4-level response scale (from 1—‘does not apply to me at all’ to 4—‘applies to me very strongly’). In Poland, a version consisting of 12 questions and a two-factor structure was proposed, which included subscales of (1) optimal regulation (OR) and (2) openness to life experiences (OL). The subscales’ reliability as determined with Cronbach’s alpha were 0.78 for OR and 0.76 for OL, and for the whole scale, it was 0.82. The internal accuracy of the scale was confirmed by comparing statistically significant correlations of individual factors with the overall results [37].

The Polish adaptation of Huber’s CRS questionnaire by Zarzycka is a measure of the position of a system of religious constructs in a human personality. It consists of five subscales as follows: an interest in religious issues (IRI), religious beliefs (RB), prayer (P), religious experience (RE), and cult (C), the latter of which is understood as the frequency and subjective meaning of human participation in religious services. The overall result is the sum of the subscale results, and it is a measure of centrality of the system of religious meaning in an individual’s personality (CoR). The scale consists of 15 items with a Likert scale to which the respondents respond by choosing between 5 and 8 possible responses. In each case, the responses are transposed to the 5-point scale (the higher the score, the greater the importance or frequency of the behaviour). The reliability of the scale, estimated using Cronbach’s alpha, was 0.82 ≤ *α* ≤ 0.90. The values of intercorrelation between positions and the scores in individual subscales indicate the accuracy of a separate theoretical construct, and the subscales can be considered homogeneous [38].

The IES-R of Weiss and Marmar in the Polish adaptation of Juczyński and Ogińska-Bulik [39] was used to measure post-traumatic stress disorder (PTSD) symptoms. Despite being based on DSM-IV, it is still one of the most popular measurement tools and enables comparisons with the results of other authors. It consists of 22 statements describing the symptoms of stress experienced in the last 7 days due to a traumatic event. It is assessed on a 5-point Likert scale (0–4). It is used to determine the current, subjective sense of discomfort associated with a specific event. It covers three dimensions of PTSD: (1) intrusion (I), the expression of recurring images, dreams, thoughts, or perceptions associated with trauma; (2) hyperarousal (H), characterised by increased vigilance, anxiety, impatience, and difficulty in focusing attention; (3) avoidance (A), which manifests as attempts to get rid of thoughts, emotions, or conversations associated with trauma. The internal consistency assessed on the basis of Cronbach’s alpha was 0.92 for the whole scale.

To measure depression (D), the authors used the PHQ-9 in the Polish adaptation of Kokoszka et al. [40], which consists of nine fundamental questions and one supplementary question. The fundamental questions concern the symptoms of depression included in the DSM-IV diagnostic criteria. The respondent marks the answers on a scale from 0 to 3, depending on the frequency of occurrence of a given symptom in the last two weeks. The PHQ-9 was very reliable, as Cronbach’s alpha is 0.88, and confirmed the significant correlation with the results of BDI (rho = 0.92, *p* < 0.001) and HRDS (rho = 0.87, *p* < 0.001).

The questions related to the COVID-19 pandemic, i.e., they were questions related to the reactions felt in connection with the experienced pandemic event. Before answering these questions, the participants completed a demographic survey. The data were collected via the Internet. The questionnaire was available in Polish. For participant recruitment, we used a snowball sampling strategy to reach the general public. However, this procedure is acceptable for exploratory research [41], which this project is. Data collection took place in March–May 2020. The completion of the survey took approximately 20 min. Participation was voluntary, and the participants did not receive any compensation.

### 2.3. Design and Construction of a Mediation Model

In the study, we were interested in the variables that, from a theoretical point of view, can mediate between mental resilience and the desired results in terms of stress resistance (PTSD). Because the Huber model is an application of Kelley’s cognitive constructivism [42] to examine religiosity, which combines the theory of religiosity with the theory of personality, this study combined it with the concept of ego-resiliency. Awareness of the fact that dealing with stress can also be done beyond the involvement of religious factors does not, of course, allow religiosity to be accepted as the sole criterion, but it was assumed that it significantly contributes to an effective process of dealing with a difficult situation by mediating the immunogenic effects of ego-resiliency. According to Pargament’s theory [29], when it comes to the hypothesis being tested, multidimensional religiosity was treated as a mediator between the psychological resource in the form of ego-resiliency and the severity of symptoms of COVID-19-related PTSD and depression. Due to the fact that the single-factor version of the tool has been shown to be significantly less effective than the two-factor version, it was decided to use the two-factor version and divide the results into two subscales (The root mean square error (approximately RMSEA = 0.069) indicates that the two-factor model is acceptable (i.e., 0.05 ≤ RMSEA ≥ 0.08). The additional factor (CFI = 0.909) indicates a fairly good level of model fitting. Although it is not above 0.95, it exceeds the limit of 0.90 and has a much higher value compared to the CFI for the one-factor solution [37]).

Mediation models are currently an important aspect of research work, as they can foster a higher order among the numerous findings concerning the predictors of mental health. It seems that learning about the mechanisms of creating and using resistance resources will, over time, allow implications to be formulated for the practice in the area of psychological assistance provided to people struggling with stress [5]. It is also particularly important in the face of the announced long-term psychological consequences of COVID-19 in most countries of the world.

### 2.4. Statistical Analysis

First, we calculated the mean and SD of the variables. Next, we performed statistical hypothesis testing analyses, in all cases adopting two-tailed *p* < 0.05 as the significance threshold. Tests included the Pearson correlation and multiple regression. The mediation model was then verified. IBM SPSS was used to analyse the data.

## 3. Results

### 3.1. Correlation Analysis

Correlations were assessed to determine the bivariate relationships between all variables (Table 2).

In the study a statistically significant negative weak correlation between optimal regulation and depression was obtained (r = −0.204; *p* < 0.01). Analysis of the relationships between openness to life experiences and depression, and between the ego-resiliency and PTSD components did not show any significant correlations. By analysing the results obtained in the study of the relationship between the components of centrality of religiosity and the components of PTSD, significant negative relationships were identified. The interest in religious issues had a statistically significant relationship with hyperarousal (r = −0.188, *p* < 0.01) and avoidance (r = −0.156, *p* < 0.05). The same features of the relationship were observed between religious beliefs and hyperarousal (r = −0.141; *p* < 0.05), prayer and hyperarousal (r = −0.188; *p* < 0.01), prayer and avoidance (r = −0.207; *p* < 0.01), as well as cult and hyperarousal (r = −0.174; *p* < 0.01) and avoidance (r = −0.178; *p* < 0.01). Overall centrality of religiosity was negatively correlated with hyperarousal (r = −0.161; *p* < 0.05) and avoidance (r = −0.153; *p* < 0.05). Additionally, there was a significant negative correlation between the overall result of PTSD and the components of centrality of religiosity: interest in religious issues (r = −0.167; *p* < 0.05), prayer (r = −0.171; *p* < 0.01), and cult (r = −0.150; *p* < 0.05). Following the components of the variables, there was also a significant negative relationship between overall centrality of religiosity and PTSD (r = −0.132; *p* < 0.05). Similar relationships were also observed between some components of centrality of religiosity and depression. In the studied group (N = 227), interest in religious issues (r = −0.169; *p* < 0.05), prayer (r = −0.191; *p* < 0.01), religious experience (r = −0.164; *p* < 0.05), and cult (r = −0.156; *p* < 0.05) were negatively and poorly correlated with depression. Overall centrality of religiosity (r = −0.177; *p* < 0.01) was negatively correlated with depression.

### 3.2. Regression Analysis

To investigate the unique interactions and contributions of the COVID-19-related predictors to depression and PTSD, we conducted a set of regression analyses (Table 3).

Predictors of depression. A multiple regression analysis carried out to verify Model 1, in which depression was the explanatory variable and the components of ego-resiliency were the predictors, showed that optimal regulation is an important predictor (=−0.204, *p* < 0.01). The proposed model proved to fit well with the data (F (1.221) = 9.740; *p* < 0.001) and explained 4.2% of the variance of the dependent variable (R^2^ = 0.042). A regression analysis was also conducted to verify Model 2, in which depression was the explanatory variable and the components of centrality of religiosity were considered the predictors. The four C-15 subscales made a significant contribution to the regression equation by explaining the extent of depression. The analysis showed that the important predictors are interest in religious issues (β = −0.169, *p* < 0.05), prayer (β = −0.260, *p* < 0.01), religious experience (β = −0.164, *p* < 0.05), and cult (β = −0.156, *p* < 0.05). The proposed model turned out to be fit the data well and explained variances of the dependent variable from 2.4% in the case of cult to 3.7% for the prayer predictor.

The overall result of centrality of religiosity also proved to be an important predictor of depression (β = −0.177, *p* < 0.01) in Model 3. This model can be described with the line y = 9.50 − 0.06x, and 3.1% of the variance of the dependent variable was explained by centrality of religiosity.

Predictors of PTSD. In the next step, a multiple regression analysis was conducted to verify Model 4, in which the components of the PTSD variable (intrusion, hyperarousal, and avoidance) were the explanatory variables, and the components of centrality of religiosity were the predictors. The four C-15 subscales made a significant contribution to the regression equation by explaining the extent of hyperarousal. The analysis showed that the important predictors for hyperarousal were interest in religious issues (β = −0.188, *p* < 0.01), religious beliefs (β = −0.141, *p* < 0.05), prayer (β = −0.188, *p* < 0.01), and cult (β = −0.174, *p* < 0.01). The proposed model turned out to fit the data well, and explained variances of the dependent variable from 2.0% in the case of religious beliefs to 3.5% for two predictors: prayer and interest in religious issues.

The analysis further showed that important predictors for avoidance are interest in religious issues (β = −0.156, *p* < 0.05), prayer (β = −0.207, *p* < 0.01), and cult (β = −0.178, *p* < 0.01). The proposed model turned out to fit the data well and explained variances of the dependent variable from 2.4% in the case of interest in religious issues to 4.3% for the prayer predictor.

### 3.3. Path Analysis

A typical situation in which moderators are sought is the presence of weak dependencies. Thus, we conducted regression analyses to test whether the strength of the effect of ego-resiliency on the level of psychopathological reactions depended on religiosity. Step 1 and Step 2 used a simple regression analysis, whereas Step 3 used a multiple regression analysis.

**Figure 2:**
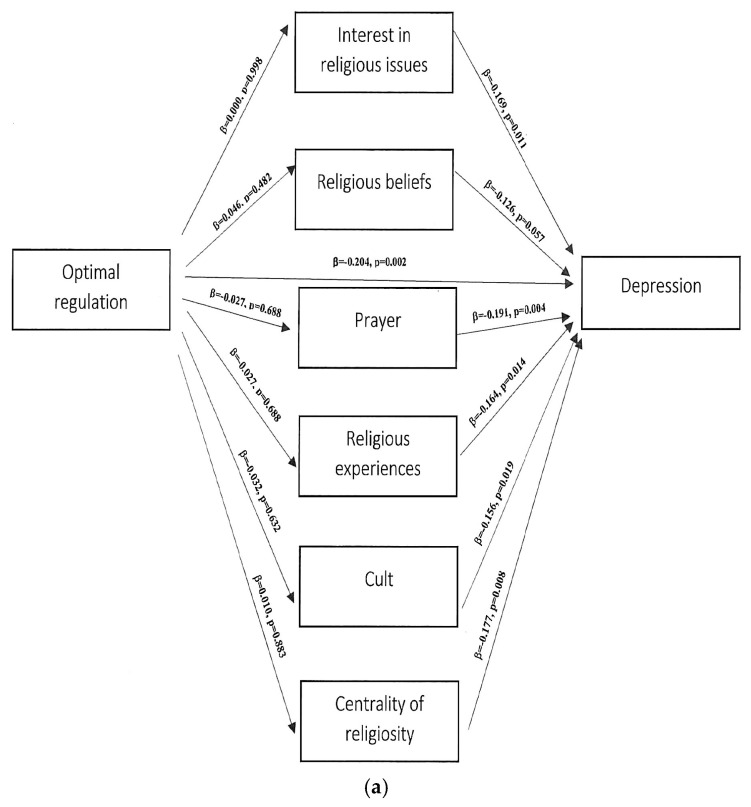

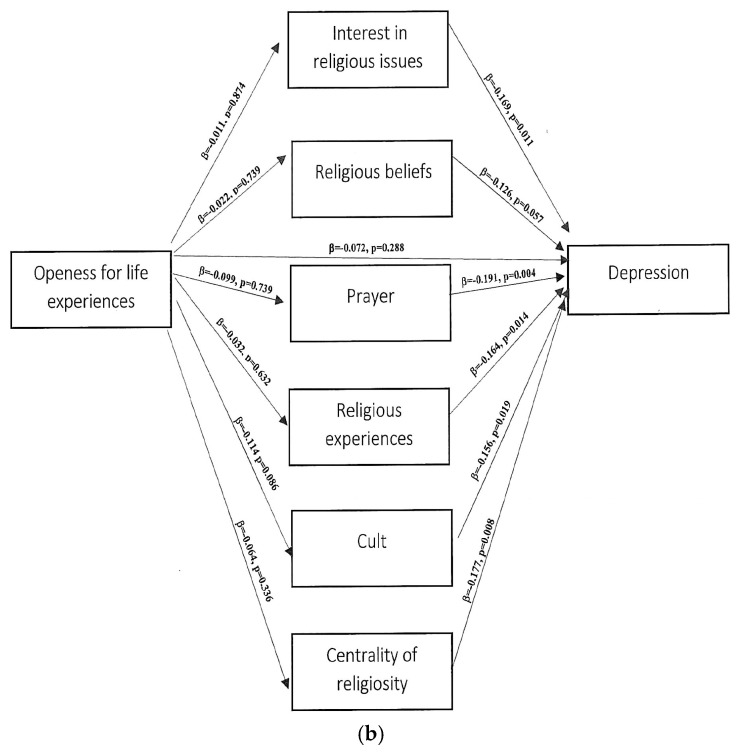
(**a**,**b**) Path analytical model of the inter-relationship between the study variables ego-resiliency, religiosity, and depression.

**Figure 3:**
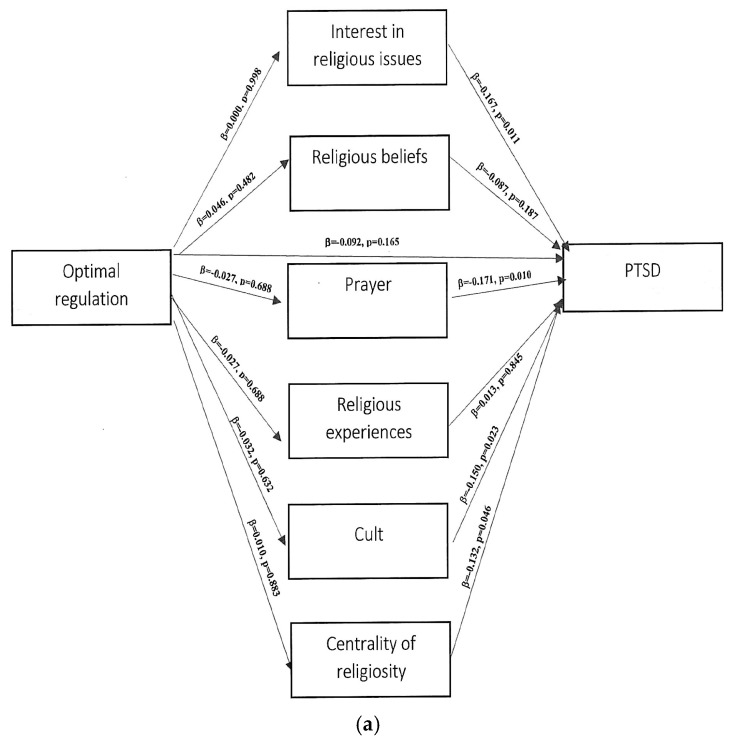

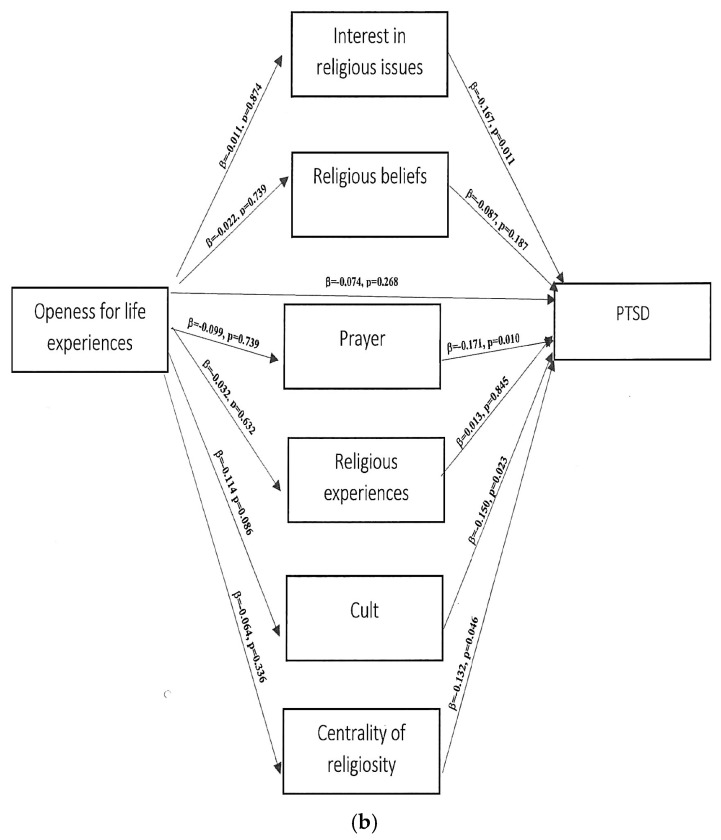
(**a**,**b**). Path analytical model of the inter-relationship between the study variables ego-resiliency, religiosity, and PTSD.

The above analysis did not confirm any significant relationship between the components of ego-resiliency, the mediator, and the dependent variable, depression (Figure 2a,b). The analysis also did not confirm any significant relationship between the components of ego-resiliency, the mediator, and the dependent variable, PTSD severity (Figure 3a,b).

### 3.4. Mediation Analysis

For the mediation analysis, it was tested whether there was a significant regression correlation between the independent variable (ego-resiliency components), the mediator (centrality of religiosity), and the dependent variables (depression and PTSD and its components). Although there is no basis in the above research model to verify the hypothesis while assuming the mediating nature of centrality of religiosity using mediation analysis, a Sobel test was conducted for individual variables to definitively confirm or rule out the mediating role of the intermediary variable [43].

The mediation analysis did not reveal an intermediary role for the components of religiosity between optimal regulation and depression. All of the results of the Sobel test were statistically insignificant, thus confirming the absence of a mediating effect between the research variables (Table 4).

## 4. Discussion

The analyses carried out showed three findings. First, ego-resiliency (optimal regulation, but not openness for life experiences) was a predictor of the severity of depression associated with the COVID-19 pandemic in the study group, but not of the PTSD symptoms. Second, religiosity was a predictor of the severity of depression (relevant components include interest in religious issues, prayer, religious experience, and cult) and of two PTSD symptoms: hyperarousal (interest in religious issues, religious beliefs, prayer, and cult) and avoidance (interest in religious issues, prayer, and cult); there were no effects regarding intrusions (partial confirmation of H1). In all cases, both optimal regulation and the described elements of religiosity had a mitigating effect on the severity of psychopathological reactions associated with the pandemic situation. Third, we assumed that the religiosity construct system would not operate as a mediator in the analysed relationships (rejection of H2). Religiosity therefore did not enhance the protective effect of ego-resiliency on levels of depression and PTSD associated with the COVID-19 pandemic. It is worth noting that in our study, although both resources analysed played an individual protective role against some of the burdens, among the relationships analysed, the vast majority did not exceed 0.2, meaning that despite some significance, they had little strength. We emphasise that we treat the obtained results with caution. Therefore, our study did not provide clear evidence for a definitely immunogenic role of the analysed resources in coping with pandemic-related psychopathological reactions, although both correlation and regression analyses indicate that they had a significant, albeit weak, protective effect. This distinguishes our results from those of other studies, which have confirmed them quite clearly.

In the literature, resilience processes are known as protective factors in dealing with adaptive problems, e.g., in socialisation [44], but also diseases [45], especially depression [46,47,48]. Research on ego-resiliency has also shown its clear protective activity in dealing with PTSD resulting from catastrophic events [49,50,51]. According to Block and Block [2], ego-resiliency is linked not only to the ability to respond to a changing situation in an adaptive manner, but also to the ability to mobilise oneself after traumatic experiences, as confirmed in previous studies [52,53]. The results that we obtained, i.e., the absence of any effect of ego-resiliency on the severity of PTSD symptoms, is somewhat surprising in this context. It seems that this may be related to the fact that the protective mechanism underlying ego-resiliency may be closely related to the ability to regulate negative effects [54], which were significantly heightened during the pandemic.

The results suggest that religiosity can be a protective factor that decreases the intensity of psychopathological reactions (depression and PTSD) associated with the COVID-19 pandemic. This is also confirmed by the results of studies on the positive role of religiosity in dealing with stress caused by the COVID-19 pandemic [55,56,57,58,59], even though the essence of this effect was not fully explained [37].

The aforementioned phenomenon has been repeatedly confirmed in studies, to be a source of hope and direction as well as a tool for prioritization according to a specific vision of life. [24,60,61,62,63,64,65,66,67,68,69,70]. A unique report from Chen et al. [71] examining the relationship between religiosity (specifically, participation in a religious service) and the so-called risk of ‘death from despair’ (related to alcohol poisoning, drug overdose, and suicide) among US health workers on a huge sample of around 100,000 respondents suggested that these phenomena are negatively correlated. However, earlier studies confirming the suppressive effect of religion on the experienced stress [45,72,73,74,75,76] did not show mechanisms triggering a typical resistance effect. There are also many studies suggesting that religiosity can play an important role in dealing with cancer, mental problems, and especially in dealing with depression, other mental disorders (e.g., psychoses), and in fighting the negative effects of somatic diseases [77,78,79,80,81,82,83,84,85,86,87,88,89]. This especially includes the works of Miller et al. [90,91], Svob et al. [92,93], and De Berardis et al. [94], which proved the suppressive effect of religiosity on depression in family transmission and suicidal behaviours (mainly among children). A lack of religious involvement was indicated as a factor associated with a positive feedback loop with depression [95,96], including during the COVID-19 pandemic [58]. The results therefore support Pergament’s theory [97] concerning the influence of religion, pointing to religion as an important philosophical orientation affording a better understanding of the world, which makes reality and suffering understandable and bearable. Faith would then act as a compensatory mechanism to re-establish a form of control (albeit illusory) and thereby reduce stress [98]. This perspective suggests that the sense of loss of control may mediate the impact of religious beliefs on stress. Anxiety, particularly as induced by a threat, has been suggested as another potential moderating variable of the effect of religiosity on psychopathological outcomes that should be considered in future studies. Psychological ego-resiliency is also associated with a reduction in negative coping strategies such as preoccupation with anxiety.

Despite the suggestion of an immunogenic nature within the centrality of religiosity in the face of psychopathological reactions during the COVID-19 pandemic, one should agree with the suggestion that the specific mechanisms underlying its protective health effects remain incomprehensible [93,99,100,101,102]. Its function seems to be closer to maintaining stability rather than plasticity, serving the need to keep the personality system unaffected by external threats. Our research ruled out its mediating relationship with ego-resiliency, which means that it acts as a direct mechanism. It is therefore necessary to agree with Zarzycka that the centrality of the religious construct system implies a probability that it functions autonomously in the configuration of other personal construct systems [38]. Religiosity may also be linked to the factor of so-called a priori beliefs, which, according to Mancini and Bonanno [103], are one of the predictors of resistance. Although Huber [104] argues that religiosity can penetrate functioning of various psychological variables [105], ego-resiliency was not such a variable in our study. A cautious explanation for this may be the age of the subjects: relatively young people, whose religiosity differs from that of their elders [106]. Therefore, religiosity may provide them with values and norms that are helpful in mobilising other resources [107,108,109,110]. It may also be worth revisiting the concept of mature religiosity (based on a genuine relationship with God), as the specific ‘functional isolation’ (lack of interaction effect) between religiosity and ego-resiliency may be a symptom of its absence [111]. It also seems that, according to Huber’s categorisation, the majority of respondents can be characterised as exhibiting low to moderate levels of religious commitment (the mean indicates the subordinate position of the religious meaning system in the personal construct system), which may be related to the lack of an activating role of religiosity in the studied relationship with ego-resiliency. Huber assumed that the high centrality of the religious construct system has a wide influence on other personal construct systems. Consequently, it influences human behaviour and experiences. Thus, if the religious construct system occupies a subordinate position in the individual’s personal system, then the influence of religious content on other psychological systems is weaker [104]. This may mean that only the centrality of religiosity would promote its effective protective effect. Our research indicates that in addition to the centrality of religiosity in the individual’s system of personal constructs, it may be worth considering something else, e.g., his or her image of God, religious emotions, attitude of religious gratitude, committed versus consensual styles of religiosity— [112], or type of bond with God [23]. This refers to an inadequate religious coping perhaps indicating a questionable relationship with God, feeling abandoned or punished by God, or the lack of a secure attachment to God based on genuine trust, or combinations of these [60,113,114,115,116]. To test this, one would need to examine emotions associated with God’s involvement in the pandemic and feelings, e.g., anger, abandonment or punishment by God, or fear that it may reflect the work of the devil [117,118]. Perhaps we should recall here the concept of crisis religion, which consists mainly of prayer and lacking in deeper engagement [119]: it was indeed prayer which in our study was the strongest predictor of pandemic-related psychopathological reactions. This evokes a reflection that it is precisely the role of prayer worth testing in the future research.

The present set of mediators did not play a significant role in psychological functioning. Though this is one of the first studies to provide a mediational analysis of the role of ego-resiliency and religiosity on health outcomes, the data are cross-sectional and therefore must be appropriately interpreted.

## 5. Limitations

The results obtained should be treated as preliminary due to the limitations of the research project. Unprecedented situation. The COVID-19 pandemic was an unprecedented phenomenon [120]. Caution is required in exercising any comparative studies with other pandemics i.e., SARS 2003, H1N1 2009, and Ebola 2014, as they did not have such a wide range as COVID-19). COVID-19 is a pandemic unlike anything else. In our research, we were able to examine not only the reactions to the pandemic itself, but also the reactions to lockdown, the results of sudden lifestyle changes, the effects of dramatic media reporting, and so on. Another limitation is that our study focussed on a specific time period: total lockdown and home quarantine. The consequences of staying constantly in the home environment may have distorted the course of some emotional processes.

Method of data collection. The sampling method and data were compiled online. Although online research is a recognized standard today, it is important to emphasize its well-documented limitations, which should be considered when interpreting the results. Some variables, such as the experience of other stressful events in the participant’s life, were not measured; however, they might have had an impact on the results.

Group size and proportions. The larger proportion of women in volunteer studies on the psychological effects of the COVID-19 pandemic is the standard rather than the exception [21,121,122,123,124,125,126], which does not mean that we are unaware of the limitation that this situation causes. The number of respondents we obtained was generally low, although sufficient to test the formulated hypotheses. We did not consider it appropriate to maximise the sample size at this stage of the research, which largely relies on a preliminary analysis of different populations and various aspects of resilience and religiosity models. Furthermore, the completion of the study was necessitated by the decision of the state authorities to end the home quarantine; further recruitment was not advisable, as it would have recruited people in a completely different psychosocial situation.

Limitations of research tools. First, the use of self-report measures that totally rely on the honesty of the individual that is reporting is inherently limiting. Second, assessing religiosity via self-report is a method vulnerable to issues such as virtue signalling, social desirability, and memory biases [127]. It is also worth adding that recent studies suggest that the used ER89 tool is particularly useful for measuring plasticity or elasticity related to flexibility [6] rather than to a two-dimensional ego-resiliency structure, which may also indirectly affect the image of the obtained dependencies or, rather, their absence.

Sociocultural context. It is worth noting that the research was carried out in Poland, thus the importance of religiosity should be related to Polish sociocultural conditions. This contextualization means that the conclusions do not have to refer to other countries or to other denominations. This context seems to be largely responsible for the meaning of religiosity. Research suggests that Poles legitimize institutional church models of religiosity to the greatest extent. However, due to the changes that took place at the beginning of the 20th century, religion is beginning to be perceived as a sphere of specific services, and religiosity is treated as partial, fragmentary acts of using these services [128,129] More recently, there is a term for so-called Polish religious syncretism or façade religiosity (https://magazynkontakt.pl/polska-wierzy/, accessed on 30 December 2021), although this term does not necessarily mean shallow faith, because even faith that does not have many points in common with doctrine can be very deep. There is also a growing discussion regarding the excessive presence of the Church in the public sphere and attempts to translate the teaching of the Church into the legal system of the state, which may not necessarily explain the conclusions obtained, but it certainly indicates an additional level of interpretation.

## 6. Conclusions

In the present study, we investigated the effects of ego-resiliency and religiosity on mental health during the COVID-19 pandemic. Further, this study examined the interaction effect of ego-resiliency and religiosity on COVID-19-related psychopathological reactions, namely, depression and PTSD. Our results showed that ego-resiliency (optimal regulation) had a negative association with depression, and religiosity had a negative association with depression and PTSD caused by the COVID-19 pandemic. The research results suggest a protective role of the studied resources in mitigating the severity of psychopathological reactions in the studied group of people. Both ego-resiliency and religiosity were associated with lowering at least some of their aspects. However, it is important to remember that the associations found were characterised by weak strength. The analysis did not provide a basis for verifying this hypothesis while assuming the mediating nature of centrality of religiosity between ego-resiliency and intensity of psychopathological reactions during the COVID-19 pandemic using mediation analyses. The studied mechanisms seem to work autonomously. It was found that religiosity is not a factor activating or reinforcing resistance to pandemic stress through ego-resiliency, although its protective effect was confirmed by regression analysis. Is it, therefore, a kind of meta-resource, influencing the perception and exploitation of other resources? Perhaps; however, it is not ego-resiliency. In the absence of mediation dependencies, the measurement error should be taken into account in addition to the possibility of there being no correlation between variables. It is also important to note that although the associations between ego-resiliency and religiosity and respondents’ emotional functioning were statistically significant, they accounted for only a fraction of the variance. This indicates that there are probably additional clinically relevant factors that were not assessed in the model that may also play an important role in functioning. Further research models in addition to the image of God and feelings towards him should explore optimism, finding purpose in life, locating control, self-efficacy, personal qualities (such as high awareness of the situation, one’s own emotions, and the behaviour of others), supporting families and communities, and age, as their effects seem to be invaluable. A detailed examination of all of these factors will require the use of many heterogeneous tools. In addition, if resilience must be assessed as processes taking place in time, these factors should be considered in terms of a system, as the system is the carrier of the process.

## Figures and Tables

**Table 1:** Basic sociodemographic characteristics.

	Female		Male		*p*		Female		Male		*p*
	n	(%)	n	(%)			n	%	n	%	
Place of residence					0.480	Marital status					0.094
village	21	12	8	14		single	90	51.4	21	36.8	
town of up to 25,000 thousand	17	9.7	4	7		married	74	42.3	30	52.6	
small town of 25–50 thousand	13	7.4	4	7		divorced	11	6.3	5	8.8	
average city of 50–300 thousand	31	17.7	5	8.8		separated	-	-	1	1.8	
large city of more than 300 thousand	93	53.1	36	63.2		widowed	-	-	-	-	
Education					0.593	Having children					0.129
primary education	2	1.1	1	1.8							
secondary education	19	10.9	10	17.5		yes	78	44.6	32	56.1	
secondary education and studying	38	21.7	11	19.3							
higher education	91	52	30	52.6		no	97	55.4	25	43.9	
higher education and studying	25	14.3	5	8.8							
Assessment of material status					0.408	Assessment of health status					0.920
very poor	1	0.6	-	-		very poor	-	-	-	-	
poor	8	4.6	3	5.3		poor	8	4.6	3	5.3	
average	65	37.1	14	24.6		average	29	16.6	10	17.5	
good	78	44.6	33	57.9		good	89	50.9	26	45.6	
very good	23	13.1	7	12.3		very good	49	28	18	31.6	

**Table 2:** Descriptive statistics and correlations among ego-resilience, religiosity, and psychopathological reactions.

		M	SD	1	2	3	4	5	6	7	8	9	10	11	12
1. OR	r	22.27	4.32	-											
	*p*			-											
2. OL	r	11.57	2.65	0.544											
	*p*			0.000											
3. I	r	11.57	2.65	−0.127	−0.054										
	*p*			0.054	0.417										
4. H	r	8.6	6.7	−0.129	−0.111	0.885									
	*p*			0.053	0.096	0.000									
5. A	r	9.25	6.03	0.015	−0.015	0.695	0.703								
	*p*			0.816	0.828	0.000	0.000								
6. D	r	6.96	6.56	−0.204	−0.072	0.560	0.619	0.368							
	*p*			0.002	0.288	0.000	0.000	0.000							
7. IRI	r	9.35	3.99	0.000	−0.011	−0.126	−0.188	−0.156	−0.169						
	*p*			0.998	0.874	0.056	0.004	0.018	0.011						
8. RB	r	11.95	4.02	0.046	−0.022	−0.062	−0.141	−0.081	−0.126	0.699					
	*p*			0.482	0.739	0.350	0.034	0.224	0.057	0.000					
9. P	r	10.41	4.5	−0.027	−0.099	−0.112	−0.188	−0.207	−0.191	0.848	0.805				
	*p*			0.688	0.137	0.091	0.004	0.002	0.004	0.000	0.000				
10. RE	r	9.29	3.93	0.069	−0.032	0.021	−0.034	−0.061	−0.164	0.733	0.757	0.809			
	*p*			0.299	0.632	0.754	0.612	0.358	0.014	0.000	0.000	0.000			
11. C	r	9.68	4.73	−0.032	−0.114	−0.089	−0.174	−0.178	−0.156	0.821	0.774	0.890	0.779		
	*p*			0.632	0.086	0.177	0.008	0.007	0.019	0.000	0.000	0.000	0.000		
12. CoR	r	50.71	19.37	0.010	−0.064	−0.083	−0.161	−0.153	−0.177	0.898	0.882	0.956	0.889	0.939	
	*p*			0.883	0.336	0.213	0.015	0.020	0.008	0.000	0.000	0.000	0.000	0.000	
13. PTSD	r	27.14	18.78	−0.092	−0.074	0.945	0.948	0.856	0.555	−0.167	−0.087	−0.171	−0.013	−0.150	−0.132
	*p*			0.165	0.268	0.000	0.000	0.000	0.000	0.011	0.187	0.010	0.845	0.023	0.046
				1	2	3	4	5	6	7	8	9	10	11	12

**Table 3:** Predictors of psychopathological reactions—results of regression analysis.

Dependent Variable	Independent Variables	(Beta)	R	R^2^	F	SR^2^
Predictors of Depression—MODELS 1–3						
Depression	Optimal regulation (N = 226)	−0.204	0.204	0.042	9.740 *	0.037
	Religiosity (N = 227)					
	Interest in religious issues	−0.169	0.169	0.029	6.603 *	0.024
	Prayer	−0.260	0.260	0.037	8.532 **	0.032
	Religious experience	−0.164	0.164	0.027	6.185 *	0.022
	Cult	−0.156	0.156	0.024	5.624 *	0.020
	Centrality of religiosity (N = 226)	−0.177	0.177	0.031	7.271 **	0.027
Predictors of PTSD (N = 230)—MODEL 4						
Hyperarousal	Interest in religious issues	−0.188	0.188	0.035	8.280 **	0.031
	Prayer	−0.188	0.188	0.035	8.253 **	0.031
	Religious beliefs	−0.141	0.141	0.020	4.559 *	0.015
	Cult	−0.174	0.174	0.030	7.094 **	0.026
Avoidance	Interest in religious issues	−0.156	0.156	0.024	5.663 *	0.020
	Prayer	−0.207	0.207	0.043	1.284 **	0.039
	Cult	−0.178	0.178	0.032	7.473 **	0.003

* *p* < 0.05; ** *p* < 0.01.

**Table 4:** Indirect effects of ego-resiliency on psychopathological reaction through religiosity.

OR/Religiosity/Depression							OL/Religiosity/Depression					
	Z	*p*	βc	*p*	βc’	*p*	Z	*p*	βc	ip	βc’	*p*
IRI	0.000	0.999	−0.204	0.002	−0.242	0.000	0.010	0.991	−0.072	0.288	−0.027	0.680
RB	−0.647	0.517	−0.204	0.002	−0.238	0.000	−0.711	0.476	−0.072	0.288	−0.109	0.099
P	0.407	0.684	−0.204	0.002	−0.247	0.000	1.610	0.107	−0.072	0.288	−0.184	0.005
RE	−0.930	0.352	−0.204	0.002	−0.234	0.000	0.932	0.351	−0.072	0.288	−0.142	0.031
C	0.476	0.634	−0.204	0.002	−0.247	0.000	1.512	0.131	−0.072	0.288	−0.157	0.018
CoR	−0.168	0.866	−0.204	0.002	−0.241	0.000	1.165	0.244	−0.072	0.288	−0.165	0.012
OR/Religiosity/PTSD							OL/Religiosity/PTSD					
IRI	0.000	0.999	−0.092	0.165	−0.134	0.039	−0.010	0.991	−0.074	0.268	−0.701	0.484
RB	−0.639	0.523	−0.092	0.165	−0.131	0.047	0.701	0.484	−0.074	0.268	−0.007	0.325
P	0.408	0.683	−0.092	0.165	−0.139	0.032	0.653	0.632	−0.074	0.268	−0.087	0.189
RE	−0.555	0.579	−0.092	0.165	−0.133	0.045	0.554	0.579	−0.074	0.268	−0.063	0.343
C	0.478	0.633	−0.092	0.165	−0.140	0.032	1.587	0.112	−0.074	0.268	−0.083	0.206
CoR	0.168	0.866	−0.092	0.165	−0.133	0.042	−1.265	0.206	−0.074	0.268	−0.073	0.264

## Data Availability

All of the data supporting the findings are contained within the manuscript. As needed, the dataset used for the present study’s conclusions can be accessed via the corresponding author on reasonable request.
